# Lithium and GADL1 regulate glycogen synthase kinase-3 activity to modulate *KCTD12* expression

**DOI:** 10.1038/s41598-019-46655-1

**Published:** 2019-07-16

**Authors:** Tai-Na Wu, Chih-Ken Chen, Chau-Shoun Lee, Bo-Jian Wu, Hsiao-Ju Sun, Chieh-Hsing Chang, Chun-Ying Chen, Lawrence Shih-Hsin Wu, Andrew Tai-Ann Cheng

**Affiliations:** 1 Institute of Biomedical Sciences, Academia Sinica, Taipei, Taiwan; 2 School of Medicine, Chang Gung University; Community Medicine Research Center & Department of Psychiatry, Chang Gung Memorial Hospital, Keelung, Taiwan; 3 Department of Medicine, MacKay Medical College; Department of Psychiatry, Mackay Memorial Hospital, Taipei, Taiwan; 4 Yuli hospital, Ministry of Health and Welfare, Hualien, Taiwan; 5 Tsao-Tun Psychiatric Center, Ministry of Health and Welfare, Nantou, Taiwan; 6 Bali Psychiatric Center, Ministry of Health and Welfare, Tamsui, Taiwan; 7 Graduate Institute of Biomedical Sciences, China Medical University, Taichung, Taiwan; 8 Department of Psychiatry, China Medical University Hospital, Taichung, Taiwan

**Keywords:** Cell signalling, Neurochemistry

## Abstract

Potassium channel tetramerization domain containing 12 (*KCTD12*), the auxiliary GABA_B_ receptor subunit, is identified as a susceptibility gene for bipolar I (BPI) disorder in the Han Chinese population. Moreover, the single-nucleotide polymorphism (SNP) rs17026688 in glutamate decarboxylase–like protein 1 (*GADL1*) is shown to be associated with lithium response in Han Chinese BPI patients. In this study, we demonstrated for the first time the relationship among lithium, GADL1, and KCTD12. In circulating CD11b^+^ macrophage cells, BPI patients showed a significantly higher percentage of KCTD12 expression than healthy controls. Among BPI patients, carriers of the ‘T’ allele (i.e., CT or TT) at site rs17026688 were found to secrete lower amounts of GADL1 but higher amounts of GABA b receptor 2 (GABBR2) in the plasma. In human SH-SY5Y neuroblastoma cells, lithium treatment increased the percentage of KCTD12 expression. Through inhibition of glycogen synthase kinase-3 (GSK-3), lithium induced cyclic AMP-response element binding protein (CREB)–mediated *KCTD12* promoter activation. On the other hand, *GADL1* overexpression enhanced GSK-3 activation and inhibited *KCTD12* expression. We found that lithium induced, whereas GADL1 inhibited, *KCTD12* expression. These findings suggested that *KCTD12* may be an important gene with respect to neuron excitability and lithium response in BPI patients. Therefore, targeting GSK-3 activity and/or *KCTD12* expression may constitute a possible therapeutic strategy for treating patients with BPI disorder.

## Introduction

For bipolar patients, lithium is the first-line choice for maintenance treatment since it can reduce the risk of relapse and suicide^1–3^. However, only 30% of patients have an excellent response to lithium with complete remission of symptoms, as has been observed for patients of European descent^4,5^. Glutamate decarboxylase–like protein 1 (*GADL1*) has aspartate 1-decarboxylase and cysteine sulfinic acid decarboxylase activities to produce β-alanine, hypotaurine, and taurine^6^. Chronic administration of lithium is found to decrease the level of taurine in the rat brain^7,8^, and the enzyme activity of GADL1 increases significantly in the presence of lithium^9^. The single-nucleotide polymorphism (SNP) rs17026688 in *GADL1* has been found to be associated with lithium response in bipolar I (BPI) patients of Han Chinese descent. Patients carrying the allele ‘T’ (i.e., CT or TT) at rs17026688 are lithium good responders, while those carrying the homozygous allele C are lithium poor responders^10^. The SNP rs17026688 T carriers have lower frequencies of recurrent episodes than non-T carriers when these patients are compared during the cumulative period of good drug adherence^11^. However, the association between rs17026688 and lithium response has not been replicated with other clinical samples from different human populations^12,13^. Therefore, the role of GADL1 in the neuropsychiatric diseases and lithium response requires further investigation.

The gene *KCTD12*, encoding potassium channel tetramerization domain containing 12, is highly associated with BPI disorder in the Han Chinese population^14^. KCTD12, one of the auxiliary GABA_B_ receptor subunits, can increase the GABA_B_ receptor expression on the cell surface and the magnitude of downstream signaling^15,16^. GABA_B_ receptors, G-protein–coupled receptors for GABA, regulate neuronal excitability in the mammalian nervous system. Thus, GABA_B_ receptors are involved in neurological and psychiatric diseases, including epilepsy, schizophrenia, depression, and anxiety^17,18^. Notably, GABA_B_ receptors, but not GABA_A_ receptors, are upregulated in the hippocampus and frontal cortex after chronic lithium treatment in rats^19,20^.

The level of GABA, the main inhibitory neurotransmitter in the central nervous system, has been reported to be low in the plasma and cerebrospinal fluid of patients with mood disorders^21–24^. In euthymic bipolar patients, the use of lithium as a mood stabilizer is found to increase the level of GABA in both plasma and cerebrospinal fluid^21,25,26^. We hypothesized that lithium or GADL1 could regulate *KCTD12* expression, and herein investigated the mechanism underlying the regulation of *KCTD12* expression by lithium and GADL1 in the human neuroblastoma cells, SH-SY5Y.

The activity of glycogen synthase kinase-3 (GSK-3) is regulated by phosphorylation. For example, phosphorylation at Ser9 (pSer9) of GSK-3β results in its inactivation, whereas pTyr279 of GSK-3α or pTyr216 of GSK-3β results in the activation of these GSKs^27^. Lithium can inhibit the activity of GSK-3, leading to release of several transcription factors into the nucleus, including cAMP response element binding protein (CREB), heat-shock factor-1, and β-catenin^28^. In this study, we addressed how lithium and GADL1 influenced the activity of GSK-3, which regulated the expression of *KCTD12* using the *GADL1* stable overexpression neuroblastoma cell line.

## Results

### The expression of GADL1, taurine, GABA, GABA B receptor 2 (GABBR2) and KCTD12 among BPI patients and healthy controls

First, we compared plasma levels of GADL1 and its catalytic product, taurine, in BPI patients and healthy controls, showing that BPI patients secreted significantly higher amounts of GADL1 than healthy controls in the plasma (Table 1). Next, we compared their secretions between T and non-T carriers at rs17026688 among BPI patients or healthy controls. In both BPI patients and healthy controls, non-T carriers had significantly higher levels of GADL1 (Supplementary Fig. S1a) and taurine (Supplementary Fig. S1b) than T carriers (Table 1).

**Table 1:** Plasma or PBMC detection in BPI patients and healthy controls.

ELISA or FACS detection	BPI vs. HC			BPI patients			Healthy controls (HC)		
	BPI	HC	p-value	T	non-T	p-value	T	non-T	p-value
GADL1 (ng/ml)	12.94 ± 4.23	11.49 ± 3.49	0.0277*	11.61 ± 3.15	14.22 ± 4.76	0.0137*	10.42 ± 3.03	12.61 ± 3.63	0.0066**
taurine (nM)	7.35 ± 2.62	9.06 ± 4.88	0.0847	6.08 ± 1.35	8.55 ± 2.98	0.0024**	6.80 ± 1.65	11.19 ± 5.92	0.0029**
GABA (pg/ml)	109.78 ± 40.95	116.62 ± 38.25	0.107	103.90 ± 31.92	105.66 ± 25.66	0.2755	118.73 ± 35.04	104.03 ± 28.17	0.0229*
GABBR2 (ng/ml)	6.89 ± 7.02	3.01 ± 5.24	<0.0001***	8.56 ± 9.01	5.07 ± 3.18	0.0335*	3.68 ± 6.40	2.29 ± 3.69	0.3635
KCTD12 ^+^ % in macrophage cells	11.93 ± 10.36	9.18 ± 6.76	0.0169*	13.74 ± 13.57	10.02 ± 4.67	0.1843	7.62 ± 4.66	10.84 ± 8.21	0.1168

Data are shown as mean (%) ± S.D. The percentage of KCTD12 expression was analyzed in the gated macrophage cells.

The differences between healthy controls and BPI patients or between T and non-T carriers among BPI patients or healthy controls were calculated by 1-tailed Mann-Whitney test (*p < 0.05; **p < 0.01; ***p < 0.001).

KCTD8, KCTD12, and KCTD16 bind to GABBR2 as part of a stable receptor complex^15,16^. We measured the plasma levels of GABA and GABBR2 among BPI patients and healthy controls. BPI patients secreted significantly higher amounts of GABBR2 than healthy controls in the plasma (Table 1). Next, we compared GABA and GABBR2 secretions between T and non-T carriers at rs17026688 among BPI patients or healthy controls. T carriers secreted higher amounts of GABA than non-T carriers among healthy controls although we found no significant difference between T and non-T carriers among BPI patients (Supplementary Fig. S1c and Table 1). T carriers (118.73 ± 35.04 pg/ml) of healthy controls also secreted significantly higher levels of GABA than T carriers (103.90 ± 31.92 pg/ml) of BPI patients, as analyzed by Mann-Whitney test with p = 0.0238 (Supplementary Fig. S1c). On the other hand, T carriers secreted significantly higher amounts of GABBR2 than non-T carriers among BPI patients, whereas no significant difference was found between T and non-T carriers among healthy controls (Supplementary Fig. S1d and Table 1). T and non-T carriers of BPI patients secreted significantly higher amounts of GABBR2 than those of healthy controls, as analyzed by Mann-Whitney tests with p = 0.0002 and p = 0.0006, respectively (Supplementary Fig. S1d).

Microglia cells, the glia cells and macrophages in the brain, can mediate neuroinflammation and express many types of neurotransmitter receptors, including GABA_B_ receptors^29,30^. Thus, we further characterized CD11b^+^ macrophage cells with respect to their KCTD12 expression. BPI patients showed a significantly higher percentage of KCTD12 expression in macrophage cells than healthy controls (Table 1). In the gated CD11b^+^ macrophage cells, the percentage of KCTD12 expression did not differ between rs17026688 T and non-T carriers among BPI patients or healthy controls (Table 1), though T carriers (13.74 ± 13.57%) of BPI patients showed a significantly higher percentage of KCTD12 expression than T carriers (7.62 ± 4.66%) of healthy controls, as analyzed by Mann-Whitney test with p = 0.0047 (Supplementary Fig. S1e). In comparison, no significant difference was found between non-T carriers of BPI patients and those of healthy controls (Supplementary Fig. S1e). Taken together with Supplementary Fig. S1d, rs17026688 T carriers of BPI patients showed a more neuro-inhibitory status, as reflected by the plasma levels of GABBR2.

### Lithium treatment increased the percentage of KCTD12 expression in SH-SY5Y neuroblastoma cells

**Figure 1:**
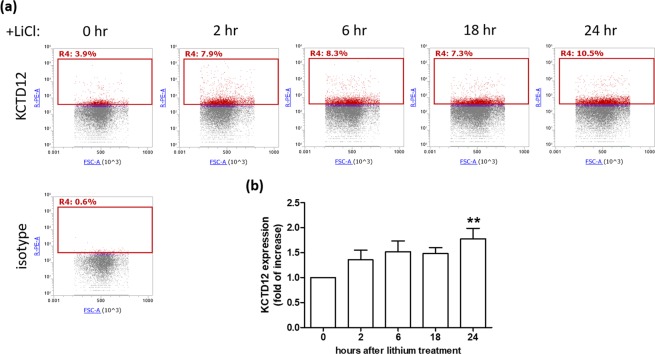
Effects of lithium treatment on SH-SY5Y cells. Human SH-SY5Y cells were treated with 20 mM LiCl for different periods of time. After trypsinization, cells were harvested for flow cytometry analysis using the primary antibody targeting KCTD12, followed by the recognition with the secondary antibody conjugated with phycoerythrin. (**a**) The percentage of SH-SY5Y cells expressing KCTD12 were shown in the R4 gate. To observe the specificity of anti-KCTD12 antibody, the isotype antibody staining was shown in the lower panel. (**b**) The time course changes of KCTD12 expression in SH-SY5Y cells were calculated and normalized to the percentage of KCTD12 expression at 0 hr from independent experiments (n = 8), followed by the statistical analysis using Dunnett’s multiple comparison test (**p < 0.01).

We examined the effects of lithium on the neuroblastoma cells. SH-SY5Y cells were treated with lithium for different periods of time. Figure 1a shows that lithium increased the percentage of SH-SY5Y cells expressing KCTD12 as time went by (0 hr vs. 24 hr: 3.9% vs. 10.5%, 2.69-fold increase). Similar results were obtained in independent experiments (n = 8), showing that 24 hr of lithium treatment increased the percentage of SH-SY5Y cells expressing KCTD12 to a 1.78-fold increase in average (Fig. 1b).

### Identification of cAMP-responsive elements (CREs) in the *KCTD12* promoter

We further explored the mechanisms for our findings that lithium increased KCTD12 expression in SH-SY5Y cells. CREB mediates the activation of cAMP-responsive genes by binding to one of the conserved CREs, TGACGTCAA, TGACG (half-site), or TGANNT(CA)^31,32^. Analysis of the *KCTD12* promoter revealed two CREs in the 869 bp upstream of the transcription start site (Supplementary Fig. S2a), suggesting that lithium-induced upregulation of *KCTD12* expression is likely mediated through CREB binding to *KCTD12* promoter. In fact, chromatin immunoprecipitation (ChIP) and luciferase reporter assays revealed that CREB could bind to the *KCTD12* promoter (Supplementary Fig. S2b) and thereby activate *KCTD12* promoter-driven luciferase activity (Supplementary Fig. S2c).

### Inhibition of GSK-3 by lithium results in the upregulation of *KCTD12* transcription

**Figure 2:**
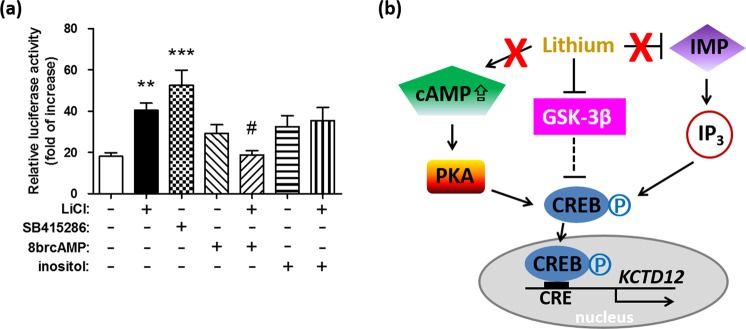
Inhibition of GSK-3β by lithium induces *KCTD12* transcription. SH-SY5Y cells were co-transfected with the CREB-EGFP plasmid and luciferase reporter construct containing *KCTD12* promoter. (**a**) At 8 h prior to harvest, 1 mM myoinositol was added in the indicated groups. At 7 h prior to harvest, cells were treated with LiCl (20 mM) or the GSK-3β inhibitor SB415286 (50 µM) in the indicated groups. At 6 h prior to harvest, cells were treated with 1 mM 8brcAMP in the indicated groups. Two days after transfection, cells were harvested to detect luciferase activity. Relative quantification of Renilla luciferase activity was normalized to firefly luciferase activity. (**b**) A proposed model describing how lithium may induce CREB-mediated *KCTD12* transcription. Through GSK-3β inhibition by lithium, but not through activation of cAMP-protein kinase A (PKA) pathway or suppression of myoinositol monophosphatase (IMP) activity, CREB enters the nucleus and binds the CRE of the *KCTD12* promoter, which activates *KCTD12* transcription. The differences at the indicated group were compared to the 1^st^ group without any treatments (**p < 0.01; ***p < 0.001) or compared to the 2^nd^ group treated with lithium only (#p < 0.05) using Newman-Keuls multiple comparison test. Data were combined from independent experiments.

A luciferase reporter assay was used for elucidating the signaling pathways underlying the lithium-induced, CREB-mediated upregulation of *KCTD12* (Fig. 2). SH-SY5Y cells were co-transfected with the CREB-EGFP plasmid and a luciferase reporter linking to *KCTD12* promoter. At 7 h prior to harvest, cells were treated with LiCl or the GSK-3β inhibitor, SB415286, in the indicated groups (Fig. 2a). Either lithium (the 2^nd^ group) or SB415286 (the 3^rd^ group) could significantly upregulate *KCTD12* promoter–driven luciferase transcription. To test whether lithium can influence cAMP-induced transcription, SH-SY5Y cells were treated with 8-bromoadenosine cAMP (8brcAMP), an analog of cAMP that has greater stability and increased membrane permeability (Fig. 2a). 8brcAMP alone (the 4^th^ group) increased CREB-mediated *KCTD12* transcription to a substantial degree (p = 0.02, as analyzed by the student t test). 8brcAMP also significantly inhibited the lithium-induced increase in the *KCTD12* transcription (the 5^th^ group), suggesting that there were no synergistic effects in the presence of lithium and 8brcAMP. In addition to GSK-3^33^, lithium salts are known to inhibit inositol monophosphatase (IMP) and thus deplete inositol in cells^34^. Therefore, the lithium-treated cells were co-treated with inositol to replenish the presumed depleted stores of inositol (the 7^th^ group in Fig. 2a). The addition of inositol did not reverse the effect of lithium on *KCTD12*-driven luciferase activity (the 7^th^ group). Inositol alone (the 6^th^ group) could induce CREB-mediated *KCTD12* transcription to a substantial degree (p = 0.007, as analyzed by the student t test). Taken together with Supplementary Fig. S2, these data indicated that lithium-induced, CREB-mediated *KCTD12* transcription acts through GSK-3β inhibition, but not through activation of cAMP-protein kinase A (PKA) pathway or suppression of IMP activity, as shown in Fig. 2b.

### Effects of *GADL1* overexpression on GSK-3 activity

**Figure 3:**
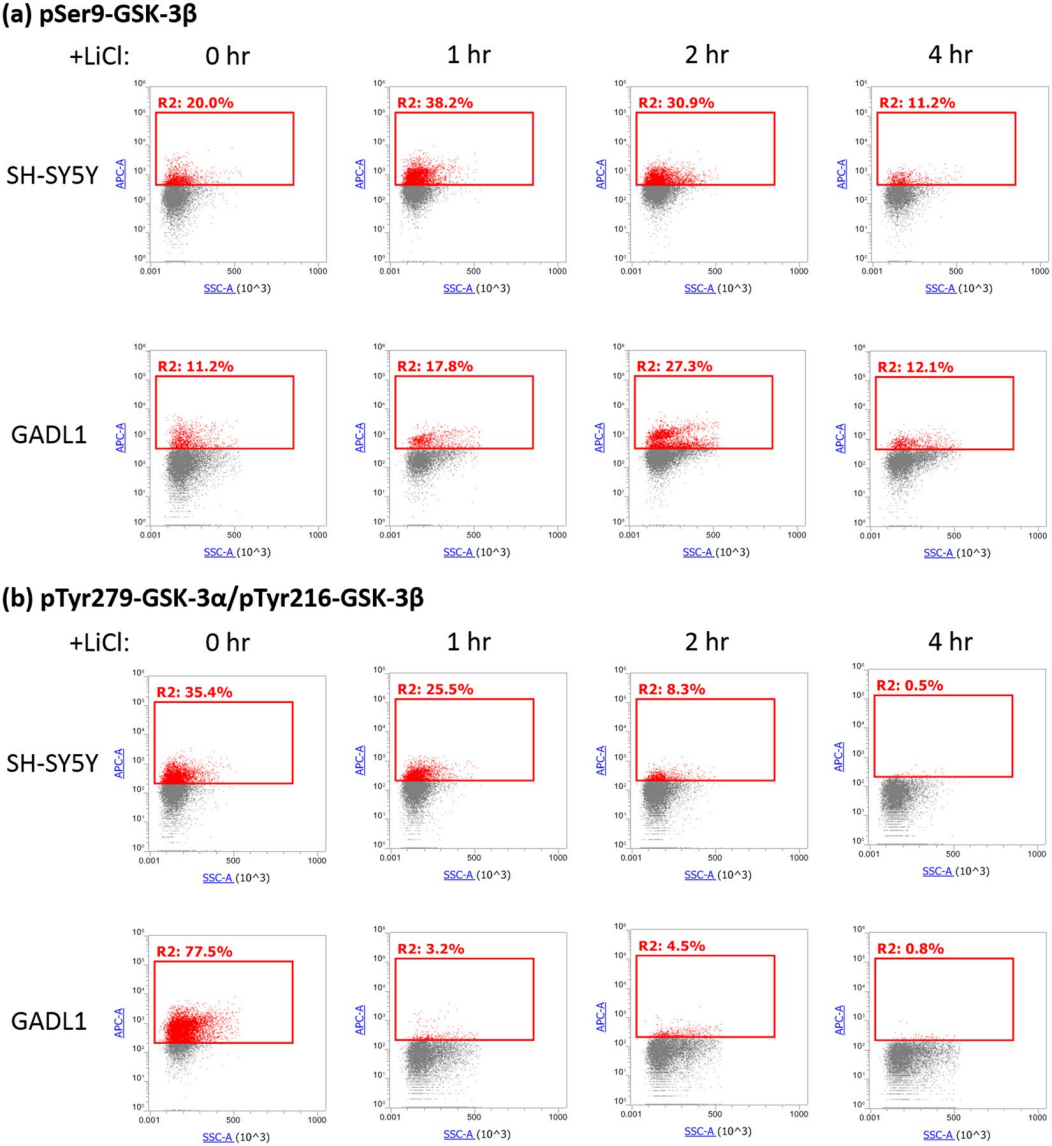
Effects of lithium treatment and *GADL1* overexpression on GSK-3 activity. SH-SY5Y cells with or without the stable overexpression of *GADL1* were plated in the medium containing 3% serum. Serum was withdrawn 48 h after cell seeding. Following serum starvation overnight, 20 mM LiCl was added for 1, 2, or 4 h. After washing out the LiCl, cells were fixed and permeabilized to stain for (**a**) pSer9-GSK-3β or (**b**) pTyr279-GSK-3α/pTyr216-GSK-3β using specific antibodies. Cells were then washed and subjected to Attune NxT flow cytometry analysis. Similar trends were observed from five independent experiments.

To test if GADL1 could affect GSK-3 activity, we established a cell clone that stably overexpressed *GADL1* using SH-SY5Y neuroblastoma cells. In the absence of lithium (0 hr), the percentage of pSer9-GSK-3β was much higher in SH-SY5Y cells (20.0%) than in *GADL1*-overexpressing cells (11.2%) (Fig. 3a), whereas the percentage of pTyr279-GSK-3α/pTyr216-GSK-3β was much higher in *GADL1*-overexpressing cells (77.5%) than in SH-SY5Y cells (35.4%) (Fig. 3b). These results indicated that *GADL1* overexpression enhanced GSK-3α/β activation but inhibited phosphorylation at Ser9 of GSK-3β, resulting in the upregulation of overall cellular GSK-3 activities. In comparison, lithium treatment increased the percentage of pSer9-GSK-3β (Fig. 3a) but decreased the percentage of pTyr279-GSK-3α/pTyr216-GSK-3β (Fig. 3b) in SH-SY5Y and *GADL1*-overexpressing cells. Hence, the effects of lithium suppressed GSK-3 activity in both types of cells.

### Downregulation of *KCTD12* mRNA level in the *GADL1*-overexpressing cells

**Figure 4:**
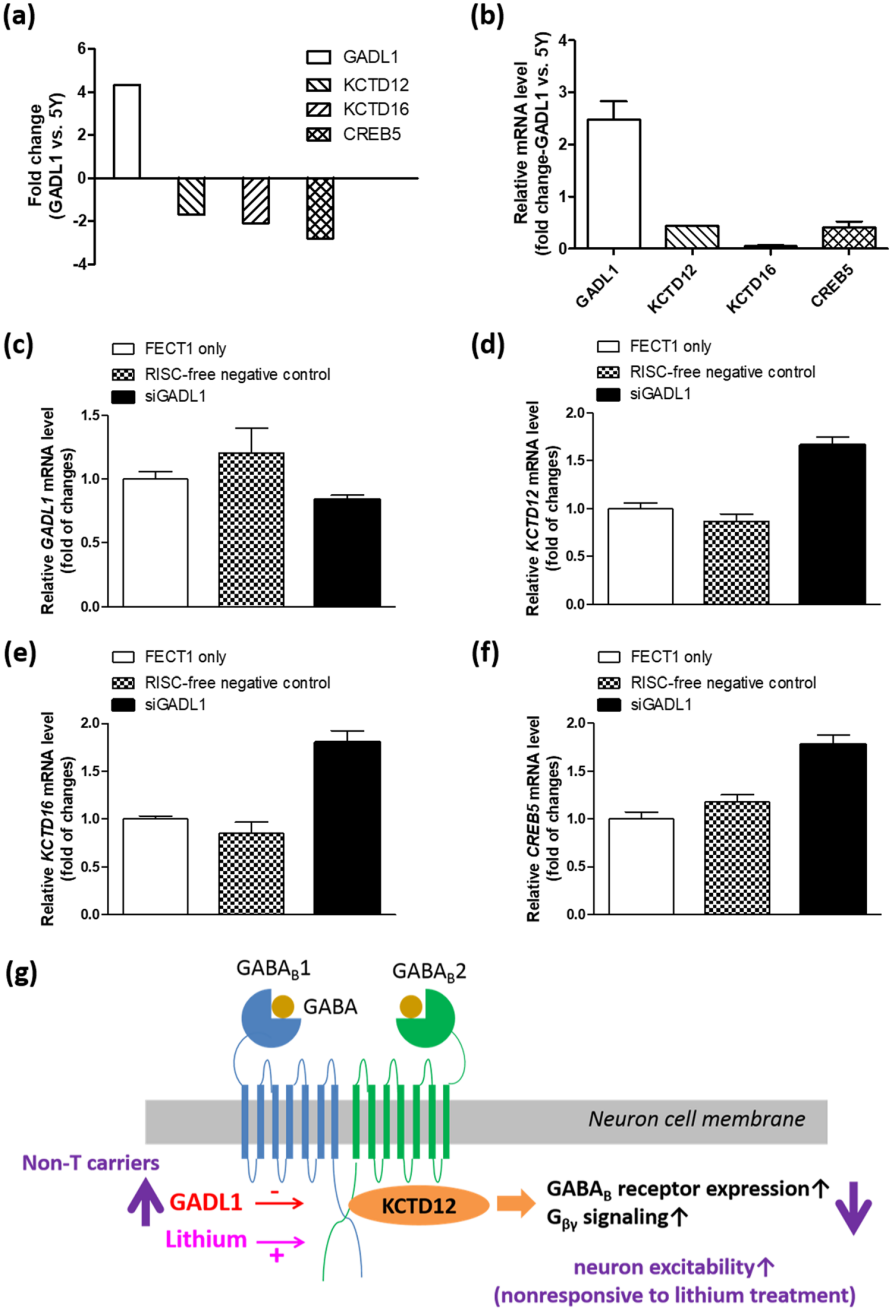
Effects of *GADL1* overexpression on expression of *KCTD* family members. (**a**) RNA expression array analyses were used to determine the level of *GADL1*, *KCTD12*, *KCTD16*, and *CREB5* mRNAs in the cells that stably overexpressed *GADL1* (GADL1) relative to the parental cell line, SH-SY5Y (5Y). (**b**) Total RNA from cells was reverse transcribed into cDNA and subjected to real-time quantitative PCR analysis for *GADL1*, *KCTD12*, *KCTD16*, and *CREB5*. Normalization to *ACTB* expression (encoding β-actin) in each sample allowed the calculation of fold-change values for *GADL1*-overexpressing (GADL1) cells relative to SH-SY5Y (5Y) cells. RNA samples for expression microarray analysis and RT-qPCR validation were prepared independently. (**c**–**f**) *GADL1*-overexpressing cells were transfected with RISC-free negative control siRNA or siRNA targeting *GADL1* (siGADL1) at 0.1 μM using DharmaFECT1 (FECT1) transfection reagent. Total RNA from cells was reverse transcribed into cDNA and subjected to RT-qPCR analysis for (**c**) *GADL1*, (**d**) *KCTD12*, (**e**) *KCTD16*, and (**f**) *CREB5*. The fold-change value for each gene was normalized to *ACTB* expression. (**g**) The proposed model for lithium nonresponsiveness in rs17026688 non-T carriers of BPI patients. KCTD12, the auxiliary GABA_B_ receptor subunit, can increase the cell-surface expression of GABA_B_ receptors and hence the magnitude of receptor signaling. Lithium upregulates KCTD12 expression and strengthens downstream G_βγ_ signaling. In contrast, GADL1 inhibits KCTD12 expression and weakens downstream G_βγ_ signaling. Non-T carriers express higher amounts of GADL1 and lower amounts of KCTD12, thereby downregulating inhibitory G_βγ_ signaling, probably contributing to the observed lithium nonresponsiveness in these patients.

Total RNA extracted from SH-SY5Y and *GADL1*-overexpressing cells was analyzed with an RNA expression array, revealing that *GADL1* was overexpressed in the stable clone as compared with the parental SH-SY5Y cell line; however, *KCTD12*, *KCTD16*, and *CREB5* were downregulated (Fig. 4a). Real-time quantitative PCR (RT-qPCR) was then performed to validate the RNA expression array data. Indeed, *GADL1* was upregulated (2.48-fold increase), whereas *KCTD12*, *KCTD16*, and *CREB5* were downregulated compared with SH-SY5Y cells (Fig. 4b).

To demonstrate a direct relationship between *GADL1* overexpression and cellular observations, we further reduced *GADL1* expression in the *GADL1*-overexpressing cell line using small interfering RNA (siRNA) knockdown. The RNA expression changes of *GADL1*, *KCTD12*, *KCTD16*, and *CREB5* after *GADL1* knockdown (siGADL1) in the *GADL1*-overexpressing cell line were examined using RT-qPCR analysis, showing that *GADL1* was knocked down to 69.5% relative to RISC-free control siRNA (Fig. 1c). As compared to RISC-free control siRNA, *KCTD12* (1.91-fold increase, Fig. 1d), *KCTD16* (2.10-fold increase, Fig. 1e), and *CREB5* (1.51-fold increase, Fig. 1f) were upregulated after siGADL1 treatment.

These data together with Supplementary Fig. S1 and Table 1, suggest a model for lithium nonresponsiveness in non-T carriers among BPI patients (Fig. 4g). Lithium upregulates *KCTD12* expression and then promotes expression of GABA_B_ receptor, which strengthens downstream G-protein–coupled (G_βγ_) signaling. In contrast, *GADL1* overexpression inhibits *KCTD12* expression. Non-T carriers express higher amounts of GADL1 but lower amounts of KCTD12, probably leading to increased neuron excitability and contributing to the nonresponsiveness to lithium treatment.

## Discussion

In this study, we found that BPI patients expressed a higher percentage of KCTD12 expression in macrophage cells than healthy controls and that rs17026688 T carriers secreted lower amounts of GADL1 and taurine than non-T carriers among Han Chinese BPI patients. Furthermore, we addressed for the first time the effects of lithium and GADL1 on the regulation of *KCTD12* expression in human neuroblastoma cells. GADL1 catalyzes the decarboxylation of aspartate, cysteine sulfinic acid, and cysteic acid to produce β-alanine, hypotaurine, and taurine^6^. In rats, chronic administration of lithium decreases the level of taurine in the brain^7,8^. This observation in rats may provide a hint for our findings that, among Han Chinese BPI patients, rs17026688 T carriers had lower amounts of secreted GADL1 and taurine than non-T carriers. Besides, we found that BPI patients secreted higher amounts of GADL1 than healthy controls in the plasma, suggesting that GADL1 might play a role in the development of bipolar disorder in the Han Chinese population. However, we acknowledged the limitation that possible confounding factors were not controlled due to the exploratory nature of this study and small sample sizes.

Monocytes can transform into microglia cells when circulating to the brain^35^. Microglia cells, the glia cells and macrophage in the brain, can mediate neuroinflammation and bear many types of neurotransmitter receptors including GABA_B_ receptors on their cell surface^29,30^. In fact, KCTD12 is highly expressed in mouse brain microglial cells^36^. In the brain, microglia cells have effects on bipolar disorder during disease development^37^. We found that BPI patients expressed higher levels of GABBR2 in the plasma and a higher percentage of KCTD12 expression in macrophage cells than healthy controls. These observations suggested that KCTD12 and/or GABA signaling pathway might be involved in the disease progression of bipolar disorder, which echoed the previous finding that *KCTD12* is a risk gene for BPI disorder in the Han Chinese population^14^.

In addition to KCTD12, T carriers secreted greater amounts of GABBR2 than non-T carriers although the plasma levels of GABA did not differ significantly between T and non-T carriers among BPI patients. Treatment with lithium has been reported to trigger an increase or no changes in the plasma levels of GABA in bipolar patients, and the amounts of rat brain GABA_B_ receptors may be increased or decreased after lithium treatment^26,38^. Among healthy controls, rs17026688 T and non-T carriers showed significant differences on the secretion of GADL1, taurine, and GABA in the plasma in this study, suggesting that the SNP rs17026688 itself had influence on the plasma levels of GADL1, taurine, and GABA secretions, which might not be related with bipolar disorder or lithium drug use.

Besides peripheral blood cells, we examined human neuroblastoma cells since most neurons in the brain express GABA_B_ receptors and at least one KCTD protein^15,39^. Moreover, GADL1 expression amounts are more in neurons than in glia cells in the human adult brain^9^. Indeed, lithium increased the percentage of KCTD12 expression in SH-SY5Y cells in our study. We hypothesized that through the upregulation of KCTD12 expression in neurons, lithium might strengthen GABA_B_ receptor signaling and reduce the neuronal excitotoxicity in the brain so as to maintain mood stability. However, this hypothesis on lithium action needs validation in the future.

We further elucidated the role of lithium in the induction of KCTD12 expression in SH-SY5Y cells. Lithium can inhibit the activity of GSK-3, leading to release of several transcription factors, including CREB, heat-shock factor-1, and β-catenin^28^. Analysis of the *KCTD12* promoter revealed two CREs in the 869 bp upstream of the transcription start site. *KCTD12* is the target gene of replication and transcription activator, a transcription activator of the gamma-herpesvirus family. This activator can form a complex with CREB, thereby activating or inhibiting CREB-response genes depending on the promoter context^40^. This evidence indirectly demonstrates that *KCTD12* is a CREB-responsive gene. Indeed, CREB could bind to the *KCTD12* promoter in both ChIP and luciferase assays. Further analysis of downstream signaling events revealed that, lithium-induced, CREB-mediated *KCTD12* transcription acts through GSK-3 inhibition.

GSK-3 contains two isoforms, alpha and beta, both of which are inhibited by lithium^41^. Our flow cytometry analysis also showed that lithium could inhibit the phosphorylation of Tyr279 of GSK-3α and/or Tyr216 of GSK-3β in SH-SY5Y and *GADL1* overexpression cells. SNPs in GSK-3β have been reported to be associated with lithium response^42^ and the age at onset^43^ in bipolar patients.* GADL1* overexpression promoted GSK-3 activation and inhibited *KCTD12* expression in our study. These findings suggested that targeting GSK-3 and/or *KCTD12* expression may constitute a possible therapeutic strategy for treating patients with BPI disorder. Actually, many mood stabilizers (e.g. valproate) and anti-psychotic drugs for treating bipolar patients have impacts on GSK-3 and related signaling events^44^.

*KCTD12* was originally identified as a susceptibility gene for BPI disorder in the Han Chinese population^14^. Here, we found that BPI patients expressed a higher percentage of KCTD12 expression in macrophage cells than healthy controls. In an earlier study, *Kctd12* knockout mice show altered emotionality, behavior, and neuronal excitability^45^. Our present study also demonstrated for the first time the relationships among lithium, GADL1, and KCTD12 in human neuroblastoma cells. Lithium increased the percentage of KCTD12 expression in SH-SY5Y cells. The effects of lithium on the induction of *KCTD12* expression were mediated though inhibition of GSK-3. Lithium-induced *KCTD12* promoter activation may contribute to the molecular mechanism underlying its therapeutic effects in the T carriers of BPI patients. In comparison, *GADL1* overexpression enhanced GSK-3 activation and inhibited *KCTD12* expression, which might weaken downstream G_βγ_ signaling. Non-T carriers expressed higher amounts of GADL1 but lower amounts of KCTD12, probably leading to more excitability in neurons and contributing to the observed lithium nonresponsiveness in these patients (Fig. 4g).

## Methods

### Study subjects

For immune endophenotype analysis, 76 BPI patients in remission (38T carriers and 38 non-T carriers) were recruited from the psychiatric departments of general hospitals and psychiatric institutions in Taiwan. A total of 60 healthy controls (31T carriers and 29 non-T carriers) were also recruited for comparisons. Their demographic characteristics are shown in Supplementary Tables S1. BPI disorder was diagnosed according to guidelines of the fourth edition of the *Diagnostic and Statistical Manual of Mental Disorders* (known as DSM-IV). Patients with other psychoses or affective disorders were excluded.

The procedures to recruit bipolar patients for this study were the same as previously described^10^. In short, psychiatric nurses and psychiatrists evaluated the study patients using a cross-culturally validated Chinese version of the *Schedules for Clinical Assessment in Neuropsychiatry* (known as SCAN)^46^ and supplemented with available medical records and reports from family members and psychiatrists. All patients were euthymic at the time of blood collection. This study was approved by the institutional review board at each participating hospital and at Academia Sinica, Taiwan.

Ethics approval for this study was approved by the ethical committee of Chang Gung Medical Foundation, Mackay Memorial Hospital, Yuli Hospital, Ministry of Health and Welfare, Tsao-Tun Psychiatric Center, Ministry of Health and Welfare, Bali Psychiatric Center, Ministry of Health and Welfare, China Medical University and Hospital, and Academias Sinica, Taiwan. Informed consents were signed by enrolled patients and healthy controls. All experiments were performed in accordance with relevant guidelines and regulations.

### Genotyping

Genomic DNA of blood samples was purified using Genomic DNA Purification kit (Qiagen, USA). Amplification-refractory mutation system (ARMS) PCR was used to tell the genotypes at rs17026688 in the beginning. The inner primers used to tell the polymorphisms at rs17026688 were 5′-CATAAAATAATTAGCATGCAAACATTGGATATTTC-3′ (forward) and 5′-CCTGTCCTCACTAATGTATGAAGATCA-3′ (reverse), giving the band products of 174 and 286 bp for C and T, respectively. The outer primers used to check the success of PCR reaction were 5′-GATCAGACACTTGACCAATCTTGTTTAA-3′ (forward) and 5′-TTTGAGGGAATATATCAAGTGAAGTGTG-3′ (reverse), giving the band product of 432 bp. Direct sequencing and TaqMan SNP probe (C__34355332_10, Thermo Fisher) were performed to further validate the genotypes at rs17026688 as described elsewhere^10^.

### Luciferase reporter assay

Using the jetPRIME transfection reagent (Polyplus), SH-SY5Y cells were transfected with a Renilla luciferase reporter plasmid carrying 869 bp of the *KCTD12* promoter (SwitchGear), a firefly luciferase reporter plasmid, and a plasmid encoding CREB1-GFP (CREB, cyclic AMP-responsive element binding protein; GFP, green fluorescent protein) or control plasmid (pEGFP-C1, Clontech). After serum starvation overnight, a specific drug or inhibitor (LiCl, myoinositol, 8-bromoadenosine cAMP (8brcAMP) all from Sigma; SB415286 from Selleckchem) was added for different periods of time. Cells were lysed in reporter lysis buffer (Promega) containing a protease inhibitor cocktail (complete, EDTA-free, Roche) 2 days after transfection. Cell lysates were prepared to measure the luminescence of Renilla luciferase and firefly luciferase using a GloMax microplate scintillation and luminescence counter (Promega). The Renilla luciferase-derived luminescence from the *KCTD12* promoter or control (Prom vector) was normalized to the luminescence measured from firefly luciferase, which accounted for differences in the transfection efficiency.

### siRNA knockdown in the *GADL1*-overexpressing cell line

*GADL1*-overexpressing cells were transfected with RISC-free negative control siRNA or siRNA targeting *GADL1* at 0.1 μM using DharmaFECT1 transfection reagent 24 hr after cell seeding, as previously described^47^. Medium was changed 24 hr after transfection. Two days post transfection, cells from sextuplicate wells were harvested and pooled for subsequent RNA extraction and reverse transcription, followed by RT-qPCR analysis for *GADL1*, *KCTD12*, *KCTD16*, and *CREB5*. The fold-change value for each gene was normalized to *ACTB* expression. These assays were done in two independent experiments.

### Statistical analysis

Statistical differences between healthy controls and BPI patients or between T and non-T carriers among BPI patients or healthy controls were calculated by Mann-Whitney tests. All statistical tests were considered significant at p < 0.05 level. GraphPad Prism 5 software was used to draw the data distribution in the figures.

## Supplementary information

supplementary information

## Data Availability

The RNA expression array datasets generated and analyzed in this study are available from the corresponding authors on reasonable request.
