# Supplementary material for: Lithium and GADL1 regulate glycogen synthase kinase-3 activity to modulate KCTD12 expression
Source: Sci Rep. 2019 Jul 16;9:10255. doi: 10.1038/s41598-019-46655-1 (PMC6635502; doi:10.1038/s41598-019-46655-1)
Supplement: Supplementary file 1 — supplementary information [file 41598_2019_46655_MOESM1_ESM.docx]

**Lithium and GADL1 regulate glycogen synthase kinase-3 activity to modulate *KCTD12* expression**

**Tai-Na Wu^1^, Chih-Ken Chen^2^, Chau-Shoun Lee^3^, Bo-Jian Wu^4^, Hsiao-Ju Sun^4^, Chieh-Hsing Chang^5,6^, Chun-Ying Chen^5,6^, Lawrence Shih-Hsin Wu^7*^, and Andrew Tai-Ann Cheng^1,7,8^***

^1^ Institute of Biomedical Sciences, Academia Sinica, Taipei, Taiwan

^2^ School of Medicine, Chang Gung University; Community Medicine Research Center & Department of Psychiatry, Chang Gung Memorial Hospital, Keelung, Taiwan

^3^ Department of Medicine, MacKay Medical College; Department of Psychiatry, Mackay Memorial Hospital, Taipei, Taiwan

^4^ Yuli hospital, Ministry of Health and Welfare, Hualien, Taiwan

^5^ Tsao-Tun Psychiatric Center, Ministry of Health and Welfare, Nantou, Taiwan

^6^ Bali Psychiatric Center, Ministry of Health and Welfare, Tamsui, Taiwan

^7^ Graduate Institute of Biomedical Sciences, China Medical University, Taichung, Taiwan

^8^ Department of Psychiatry, China Medical University Hospital, Taichung, Taiwan

*** Correspondence:** Dr. Lawrence S.H. Wu, Graduate Institute of Biomedical Sciences, China Medical University, Taichung, Taiwan and Dr. Andrew T.A. Cheng, Graduate Institute of Biomedical Sciences, China Medical University, Taichung and Institute of Biomedical Sciences, Academia Sinica, Taipei, Taiwan.

Phone: + 886-4-22052121 ext. 7732; Fax: + 886-4-22333641;

E-mail: [lshwu@hotmail.com](mailto:lshwu@hotmail.com) (Dr. L.S.H. Wu)

Phone: +886-2-2789 9119; Fax: +886-2-27823047;

E-mail: [bmandrew@gate.sinica.edu.tw](mailto:bmandrew@gate.sinica.edu.tw) (Dr. A.T.A. Cheng)

***Supplementary information***

**Supplementary Materials and Methods**

**Cell culture of SH-SY5Y cells**

SH-SY5Y cells, a human neuroblastoma line, were grown in Dulbecco's Modified Eagle Medium (DMEM)/F12 medium (1:1) (Life Technologies) supplemented with 10% fetal bovine serum (Life Technologies), 2 mM l-glutamine, 100 U/ml penicillin, and 100 μg/ml streptomycin (Life Technologies) at 37°C in a humidified incubator containing 5% CO_2_.

20 mM LiCl (Sigma Aldrich) was added for different periods of time to assess the effects of lithium. The dose of 20 mM LiCl used in the *in vitro* experiment was based on a previous study showing that lithium at that dose activated MAPK and inhibited GSK-3β in SH-SY5Y cells, with no evidence of cytotoxicity (1).

**Establishment of *GADL1*-overexpressing stable cell line**

The full-length gene *GADL1* was cloned into a vector bearing an enhanced GFP reporter and the *neo* gene from Tn5 encoding an aminoglycoside 3'-phosphotransferase, which conferred resistance to G418. SH-SY5Y cells were transfected with the plasmid encoding *GADL1* using Xfect^TM^ transfection reagent (Clontech). At 48 h post-transfection, GFP^+^ cells were sorted using a FACSAria II cell sorter (BD Biosciences). Stable cell lines were established via selection with G418 (300 μg/ml) for 2 weeks and maintained at 100 μg/ml thereafter.

**Chromatin immunoprecipitation (ChIP) assay**

ChIP was performed with a kit from Qiagen. In short, SH-SY5Y cells in 100-cm^2^ culture dishes were cross-linked in 1% formaldehyde solution (Fisher Scientific, Pittsburgh, PA). Cells were lysed and sonicated using the Bioruptor^®^ Pico sonication device (Diagenode) to shear the DNA to an average length of 300–500 base pairs (six, 8-s bursts at 50% maximum output power on ice). Samples were then pre-cleared with a slurry of protein A beads with rocking at 4°C for 1 h. Afterwards, the samples were centrifuged at 1000 × *g* for 1 min at 4°C, and the supernatant was transferred to a fresh 2.0-ml tube.

Immunoprecipitation of CREB was carried out with an antibody from Abcam (ab31387). Non-immune serum was used as a non-specific precipitator and control. After overnight immunoprecipitation on a rotator at 4°C, ChIP samples were then washed and eluted, and subsequently treated with proteinase K, Tris-HCl and EDTA. The immunoprecipitated DNA was extracted and resuspended in distilled/deionized H_2_O and subjected to real-time PCR analysis (Applied Biosystems) using primers specific for the *KCTD12* promoter (Qiagen, GPH1017718 (+)01A) or *GAPDH* (forward: 5’-CGGGATTGTCTGCCCTAATTAT-3’; reverse: 5’-GCACGGAAGGTCACGATGT-3’) as a negative control; *GAPDH* is not a target of CREB (2). The immunoprecipitated (IP) DNA was calculated to the percentage of total input DNA (2^-[Ct (IP)-Ct (input)]^ = 2^-△Ct^ = fraction of input), and then normalized to the amount of *GAPDH* detected in the presence or absence of anti-CREB1 antibody.

**Enzyme-linked immunosorbent assay (ELISA)**

Blood (10 ml) was drawn from each subject by venipuncture into a tube containing sodium citrate. The blood was immediately centrifuged at 3000 × *g* for 10 min. The plasma was collected and stored at −80°C until assayed. Plasma levels of GADL1 (MyBioSource), taurine (MyBioSource), GABA (Cloud-Clone Corp.) and GABBR2 (MyBioSource) were measured by specific ELISA kits according to the procedures supplied by the manufacturer.

**Flow cytometry analysis of KCTD12 expression**

Peripheral blood (10 ml) was collected by venipuncture and stored in sodium citrate tubes prior to analyses. Peripheral blood mononuclear cells (PBMCs) were isolated using Ficoll-Paque (GE Healthcare) density gradient centrifugation for 30 min at 1000 × *g*. The freshly isolated PBMCs were washed with 1X phosphate-buffered saline (PBS) containing 0.05% sodium azide and then treated with Fc Block solution (Miltenyi Biotec, Germany) on ice for 20 min. Next, PBMCs were stained with antibodies specific for CD11b (Biolegend) and KCTD12 (LSBio) on ice for 30 min. After washing, the secondary anti-rabbit IgG conjugated with phycoerythrin was used to detect the anti-KCTD12 antibody on ice for another 30 min. The PBMCs were washed and analyzed with a Canto II flow cytometer (BD Biosciences).

Human SH-SY5Y cells were treated with 20 mM LiCl for different periods of time. After trypsinization, cells were harvested for flow cytometry analysis using the primary antibody targeting KCTD12, followed by the recognition with a secondary antibody conjugated with the fluorescent dye. Cells were then washed and subjected to Attune NxT flow cytometry (Thermo Fisher Scientific) analysis.

**Flow cytometry analysis of cellular GSK-3 activity**

SH-SY5Y cells with or without the stable overexpression of *GADL1* were plated in DMEM/F12 (1:1) medium containing 3% fetal bovine serum. Serum was withdrawn 48 h after cell seeding. Following serum starvation overnight, 20 mM LiCl was added for 1, 2, or 4 h. After wash, cells were fixed and permeabilized using the cell signaling buffer set (Miltenyi Biotec) to stain for GSK-3β, pSer9 (Miltenyi Biotec) and GSK-3α/β, pTyr279/pTyr216 (Bioss, USA) using specific antibodies. Cells were then washed and analyzed with an Attune NxT flow cytometer (Thermo Fisher Scientific).

**RNA expression microarray analysis**

Total RNA was extracted from SH-SY5Y cells with or without the stable overexpression of *GADL1* using the NucleoSpin RNA/protein isolation kit (MACHEREY-NAGEL, Germany) as previously described (3). RNA from each cell line was subjected to single microarray chip analysis. RNA quality was determined before hybridization. Total RNA (10 µg) was used for cDNA synthesis, labeled via *in vitro* transcription, followed by fragmentation according to the manufacturer’s suggestion (GeneChip Expression Analysis Technical Manual rev5, Affymetrix, USA). Each labeled sample (11 µg) was hybridized to the GeneChip Human Transcriptome Array 2.0 (HTA 2.0) (Affymetrix) at 45°C for 16.5 h. The wash and staining steps were performed with a Fluidic Station-450, and the GeneChip HTA 2.0 was scanned with the Affymetrix GeneChip Scanner 7G. Changes in the interested gene expression in the *GADL1*-overexpressing cells relative to the parental SH-SY5Y cells were assessed with GeneSpring software (Agilent) and IPA software ([Ingenuity Pathway Analysis, Qiagen](https://www.qiagenbioinformatics.com/products/ingenuity-pathway-analysis/)). The fold changes of interested gene expression were depicted using GraphPad Prism 5 software.

**mRNA extraction and real-time quantitative PCR**

Total RNA was extracted from SH-SY5Y cells with or without *GADL1* stable overexpression using the NucleoSpin RNA/protein isolation kit and subjected to reverse transcription to yield cDNA using the reverse transcription kit (Roche). Expressions of *GADL1* (Qiagen, PPH22451A), *CREB5* (Qiagen, PPH06982A), *KCTD16* (Qiagen, PPH58103A), and *KCTD12* (5’-GATCTGGACCAGCTACACCG-3’, forward; 5’-ACCCAGCTACTACTGGAGGG-3’, reverse) were examined with SYBR Green (Qiagen) using gene-specific primers. The primers for *ACTB* (Qiagen, PPH00073G) was used for the relative quantification of *GADL1*, *KCTD12*, *KCTD16*, and *CREB5* mRNAs. Quantitative, real-time PCR was performed with an ABI 7500 system (Applied Biosystems).

**Supplementary Table S1.** **Demographic and clinical characteristics of bipolar I patients and healthy controls with rs17026688 polymorphisms.**

|  | BPI vs. HC | | | BPI patients | | | Healthy controls (HC) | | |
| --- | --- | --- | --- | --- | --- | --- | --- | --- | --- |
|  | BPI | HC | p value | T carriers | non-T carriers | p value | T carriers | non-T carriers | p value |
| Group size | 76 | 60 |  | 38 | 38 |  | 31 | 29 |  |
| Age, years (mean ± S.D) | 48.01 ± 9.88 | 31.68 ± 5.88 | <0.0001*** | 49.08 ± 9.61 | 46.95 ± 10.16 | 0.35 | 31.74 ± 6.96 | 31.62 ± 4.56 | 0.94 |
| Sex |  |  | 0.026* |  |  | 0.82 |  |  | 0.2 |
| Men | 31 (41%) | 36 (60%) |  | 15 (39%) | 16 (42%) |  | 21 (68%) | 15 (52%) |  |
| Women | 45 (59%) | 24 (40%) |  | 23 (61%) | 22 (58%) |  | 10 (32%) | 14 (48%) |  |

The statistical differences in age are calculated using two-tailed student t test, while those in sex are calculated using χ^2^ test (Pearson). T and non-T carriers are compared among BPI patients or healthy controls.

***Supplementary figures and figure legends***


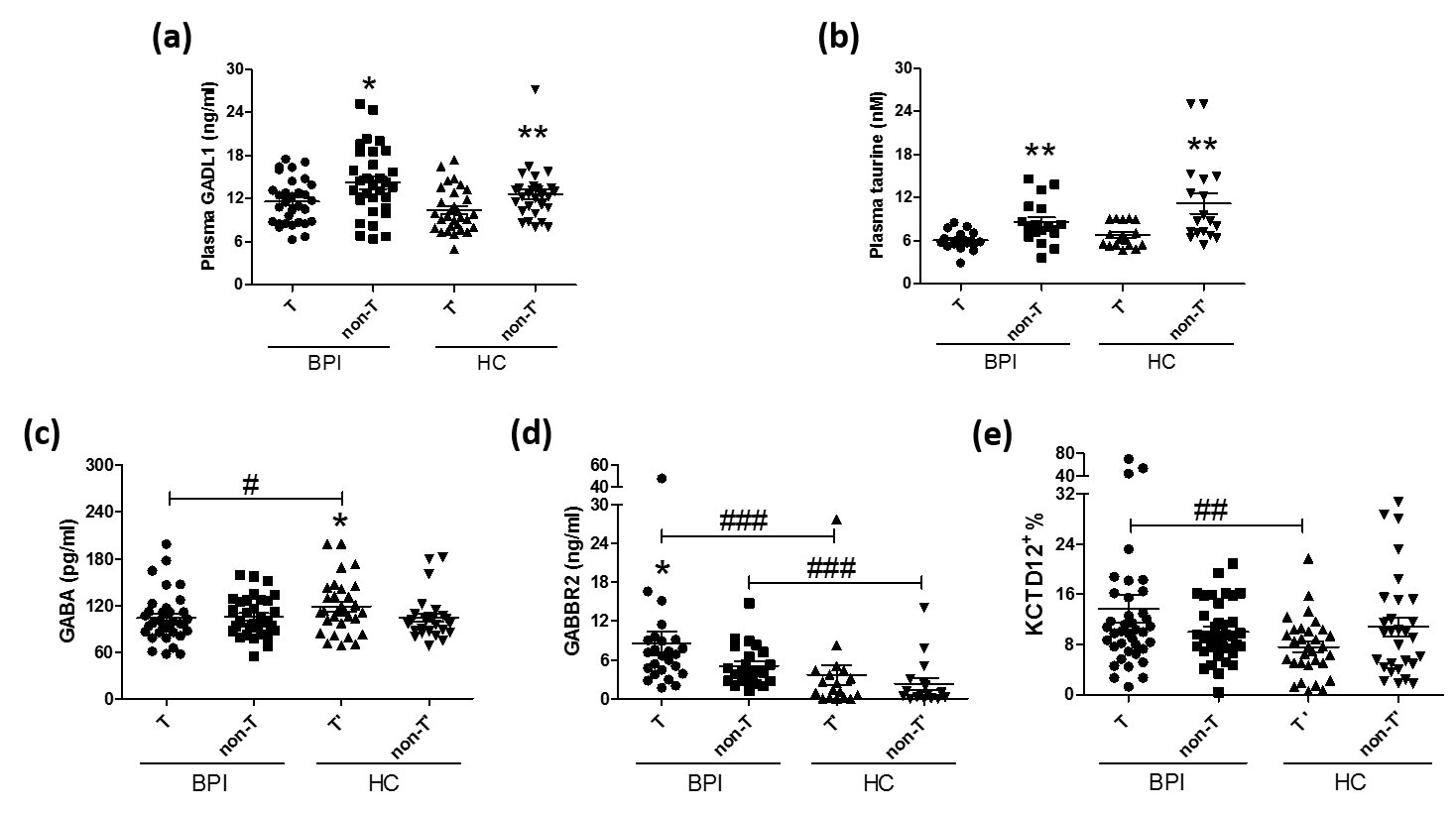


**Supplementary Fig. S1 Plasma levels of GADL1, taurine, GABA, and GABBR2 as well as the percentage of KCTD12 expression in CD11b^+^ macrophage cells among BPI patients and healthy controls**

Plasma levels of (a) GADL1, (b) taurine, (c) GABA, and (d) GABBR2 were measured in samples from BPI patients and healthy controls (HC) using specific ELISA kits. PBMCs were stained with antibodies specific for CD11b and KCTD12. (e) The percentage of KCTD12 expression in the gated CD11b^+^ macrophage cells was analyzed with flow cytometry. Statistical differences between T and non-T carriers among BPI patients or healthy controls were indicated by Mann-Whitney test (*p < 0.05; **p < 0.01). The differences between healthy controls and BPI patients were calculated by Mann-Whitney test (#p < 0.05; ##p < 0.01; ###p < 0.001). Horizontal lines were represented as mean ± SEM.

**(a)**

TGCAACTGTTGTGAGTCTAAGGGCGCGATTCAACGAAGGCTGAATGGGACCCGGAGACCG

AACGTTCCGGAGTCTTACCCCTTCACTTCGAAGTGAAGTGCCTCGAAGGCCCTGCGGGAA

GAGGGAATTGCAATTAAGAGCAGATTTGAAGCTTCATCTCATTTTGTCTCTGCAGCCAGA

AAATAAACTATCGTAAAGCATCCTACTGCGGGACACTGAGGTTTGCTTTTTGTTTCTAGG

TTTATTTATTTATTTTCCAGGCTGGCTGCAAGGCAACTTGAACAAAATCTGGCTGCGCTC

GGACTGGGGCGGCTCTTTCGTCTCTCCAGGTTTTTCGCGTCTCTCTACTTTCAGTGACAC

GACAACCAACGATGGCAGGCCGCAGCTGCACTGCTGGAAGCGCCCAGTTCCGCAGGCGGG

AGGGCCGGAGGCGGCCACGCATTTCCACTCCCGGGGCGACAGGCTTCCGAAAGTTCACGT

CCTCCTTCCAGAGTCCTTGGGTGCGGGCTGGGGCGAGAGGGGAGGGGGCGCGGTGACTGT

CCCTCCAAGGAGCTGTCTTCCGAAGCCGCCAAGTCTCGGGCGGGTCCTCTTCGGAAGAGG

GAGAAAGTTGGGCGGGACCACGGGGCGGGGCTGGTGGGTGGGGAGCGGGTGGGAGTCCGGGCGCCCCCAGCGCTCGCGAGCCGGGGTGGAGCCAGCAGCCCCGCGTGGCGTCCGCGTTTAAGATGCAAATCGAGTCCCCGCTCCCCACCTCCCCTCGCCCGGTAAGCGAGGCTCCGCCGC

CGCCCACGCCTCCTGCGCGCCACGGCACCAGAGCAGGCGGCTCTACTTAAGCAGCGCGCG

GGCCGCGACCCGGCACTCGCCTGGAGCGCGCGGGCAAGGCGGGCGGAGCGCACTGGAACT

CAAGGGGGCGCACAGCGGCGCGCTCGCACCGCTCGGCTCCGCGCGGCTCTAGGAGGTGGC

GGCGGTGGCGGTGGCGGCGGTGGCGGCGGCGGCGGCGGCGGGGCGCAGGGCTGAGCGAGCGTCCGGGTTCCGGGGCTCCGGGGAAGGCGGTTGCAGCTCCTGAGTGCGCGGGCAAGGC


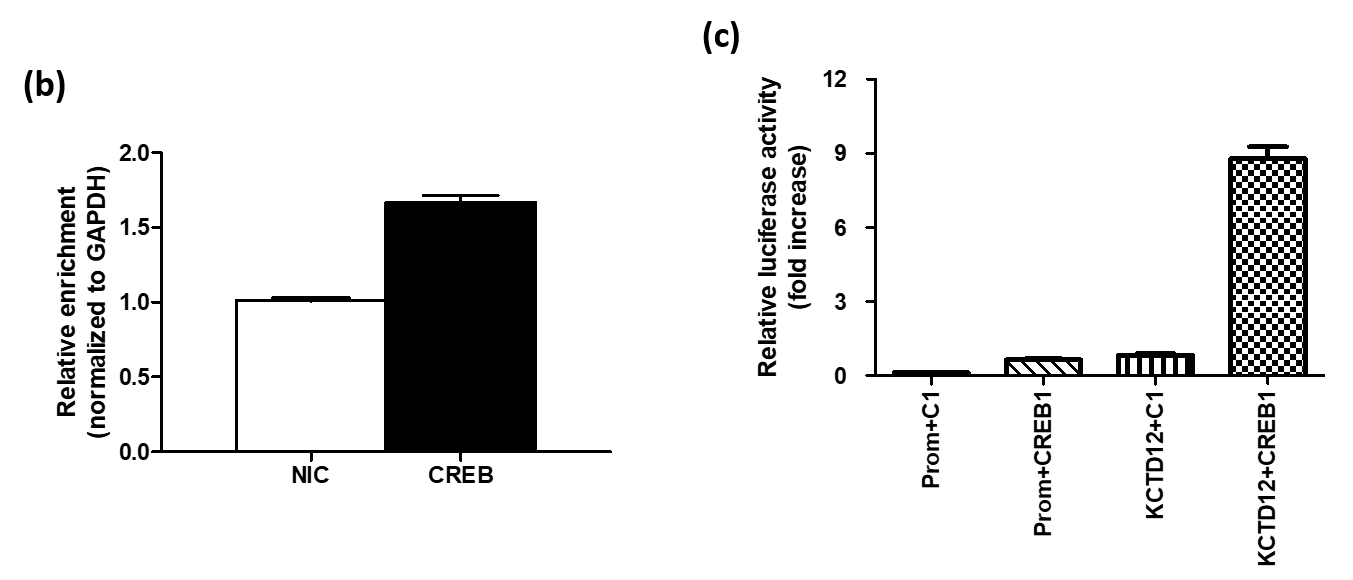


**Supplementary Fig. S2 CREB1 binding to the *KCTD12* promoter and subsequent activation of *KCTD12***

(a) 869 bp upstream of the transcription start site (framed G) was shown as the promoter region of *KCTD12* gene. The potential binding sites (cAMP-responsive elements, CREs) for CREB were underlined. (b) SH-SY5Y cells were harvested and fixed for the ChIP assay with anti-CREB1 or with non-immune serum control (NIC). The immunoprecipitated DNA was amplified using primers specific for *KCTD12* or *GAPDH*. Relative quantification was done after calculation of the percentage of total input DNA and then normalized to the amount of *GAPDH* detected in the presence or absence of anti-CREB1. Data were combined from three independent experiments as fold of increases relative to NIC and expressed as mean ± sem. (c) SH-SY5Y cells were transfected with the plasmid C1 with or without *CREB1* and the plasmid Prom carrying the Renilla luciferase gene with or without the *KCTD12* promoter. After 2 days, cells were harvested to detect luciferase activity. Relative quantification of Renilla luciferase activity was normalized to firefly luciferase activity.

**References**

1. Mai L, Jope RS, & Li X (2002) BDNF-mediated signal transduction is modulated by GSK3beta and mood stabilizing agents. *Journal of neurochemistry* 82(1):75-83.

2. Wang R*, et al.* (2006) Transcriptional regulation of PEN-2, a key component of the gamma-secretase complex, by CREB. *Molecular and cellular biology* 26(4):1347-1354.

3. Wu TN, Chen CK, Liu IC, Wu LS, & Cheng AT (2019) Effects of GADL1 overexpression on cell migration and the associated morphological changes. *Scientific reports* 9(1):5298.
